# The RING-H2 gene *LdXERICO* plays a negative role in dormancy release regulated by low temperature in *Lilium davidii* var. *unicolor*

**DOI:** 10.1093/hr/uhad030

**Published:** 2023-02-20

**Authors:** Xinyue Fan, Xiaoman Zou, Linlan Fu, Yue Yang, Min Li, Chunxia Wang, Hongmei Sun

**Affiliations:** Key Laboratory of Protected Horticulture of Education Ministry, College of Horticulture, Shenyang Agricultural University, Shenyang 110866, China; Key Laboratory of Protected Horticulture of Education Ministry, College of Horticulture, Shenyang Agricultural University, Shenyang 110866, China; Key Laboratory of Protected Horticulture of Education Ministry, College of Horticulture, Shenyang Agricultural University, Shenyang 110866, China; Key Laboratory of Protected Horticulture of Education Ministry, College of Horticulture, Shenyang Agricultural University, Shenyang 110866, China; Key Laboratory of Protected Horticulture of Education Ministry, College of Horticulture, Shenyang Agricultural University, Shenyang 110866, China; Key Laboratory of Protected Horticulture of Education Ministry, College of Horticulture, Shenyang Agricultural University, Shenyang 110866, China; Key Laboratory of Protected Horticulture of Education Ministry, College of Horticulture, Shenyang Agricultural University, Shenyang 110866, China; National and Local Joint Engineering Research Center of Northern Horticultural Facilities Design and Application Technology, Shenyang 110866, China

## Abstract

Dormancy regulation is the basis of the sustainable development of the lily industry. Therefore, basic research on lily dormancy is crucial for innovation in lily cultivation and breeding. Previous studies revealed that dormancy release largely depends on abscisic acid (ABA) degradation. However, the key genes and potential regulatory network remain unclear. We used exogenous ABA and ABA inhibitors to elucidate the effect of ABA on lily dormancy. Based on the results of weighted gene coexpression network analysis (WGCNA), the hub gene *LdXERICO* was identified in modules highly related to endogenous ABA, and a large number of coexpressed genes were identified. *LdXERICO* was induced by exogenous ABA and expressed at higher levels in tissues with vigorous physiological activity. Silencing *LdXERICO* increased the low-temperature sensitivity of bulblets and accelerated bulblet sprouting. *LdXERICO* rescued the ABA insensitivity of *xerico* mutants during seed germination in *Arabidopsis*, suggesting that it promotes seed dormancy and supporting overexpression studies on lily bulblets. The significant increase in ABA levels in transgenic *Arabidopsis* expressing *LdXERICO* indicated that *LdXERICO* played a role by promoting ABA synthesis. We generated three transgenic lines by overexpressing *LdICE1* in *Arabidopsis thaliana* and showed that, in contrast to *LdXERICO*, *LdICE1* positively regulated dormancy release. Finally, qRT–PCR confirmed that *LdXERICO* was epistatic to LdICE1 for dormancy release. We propose that *LdXERICO*, an essential gene in dormancy regulation through the ABA-related pathway, has a complex regulatory network involving temperature signals. This study provides a theoretical basis for further exploring the mechanism of bulb dormancy release.

## Introduction

As a typical bulbous flowering genus, lily constitutes an important portion of the global flower market because of its widespread application and high economic value [1, 2]. *Lilium* L. has evolved natural dormancy, and the degree of dormancy varies among different species and varieties [3]. It has been recognized that 4°C treatment is the most effective method to release lily bulb dormancy. Dormant bulbs usually need to be stored at low temperature for a certain period of time before they can complete growth, development, and flowering in the next life cycle [4]. Low temperature can release bulb dormancy by affecting carbohydrate metabolism balance, hormone balance, and peroxidase activity [3, 5–7]. However, to date most of the relevant existing studies have focused on the morphological, physiological, and biochemical changes during dormancy release, and basic research on this physiological process is relatively scarce. Therefore, to overcome the bottleneck of technological progress in the lily industry and to promote the development of the industry, it is highly important to explore the biological basis of lily dormancy, to screen and explore the key genes involved in this process, and to explore the regulatory functions that reveal the molecular mechanism underlying lily bulb dormancy.

Plant hormones are small signaling molecules produced naturally in plants and play an important role throughout the whole plant life cycle [8]. Abscisic acid (ABA) is a key plant hormone that controls the stress response, dormancy, flowering, and development in plants [4, 9, 10]. A large number of studies have shown that endogenous ABA levels increase significantly when plants enter dormancy and decrease significantly when dormancy is released, as has been shown in peach (*Prunus persica*) [11], pear (*Pyrus pyrifolia*) [12], cherry (*Prunus avium*) [13], lily (*Lilium davidii* var. *unicolor*) [4], gladiolus (*Gladiolus hybridus*) [9], etc. In addition, studies on the relationships between ABA levels and degree of dormancy in seeds and buds of perennial plants have shown that ABA is an indispensable hormone in regulating dormancy [14, 15] and is regulated by environmental signals [16, 17]. Moreover, the photoperiod can increase ABA content, and prolonged low temperature is usually the main cause of decreased ABA levels [18].

RING domain-containing proteins are a class of E3 ubiquitin ligases that are widespread in eukaryotic plants. Since their discovery, many RING-type E3 members have been shown to participate in various ABA-related reactions [19]. Among the different types of known RING domain-containing proteins, two typical C3H2C3 (RING-H2) and C3HC4 (RING-HC) RING domains are the most abundant [20]. Among the RING domain-containing proteins, XERICO, which encodes a RING-H2-type protein, has been widely reported to be involved in the abiotic stress response of plants [21–24]. In the early stage of seed germination and seedling growth, *XERICO*-overexpressing *Arabidopsis* plants were found to be sensitive to salt stress, osmotic stress, and ABA treatment. Further studies revealed that XERICO interacts with the AtTLP9 protein in the ABA signaling pathway, suggesting that XERICO may affect the drought tolerance of plants by regulating ABA signaling [21]. Under abiotic stress, the upregulation of *PtXERICO* in poplar reduced transpiration water loss and ion leakage, led to significant accumulation of ABA levels in cells, and indirectly increased the expression of *PtNCED3*, a key rate-limiting enzyme-encoding gene involved in ABA biosynthesis, which resulted in strong tolerance of drought stress [23]. Overexpression of the *XERICO* homologue *OsRHP1H2* in rice increased drought and salt tolerance by increasing ABA content [22]. In maize, *ZmXerico1* and *ZmXerico2* had similar functions, although their sequences are <30% similar to that of *AtXERICO*. The overexpression of *ZmXerico1* and *ZmXerico2* resulted in an increase in ABA levels, and at the same time, the levels of diphaseic acid and phaseic acid in ABA degradation products showed a downward trend. The results confirmed that ZmXerico1 and ZmXerico2 proteins improved the drought tolerance of maize by regulating ABA metabolism [20]. Although the function of *XERICO* in the plant stress response has been widely confirmed, its function in plant dormancy has not been clearly elucidated.

This study showed that ABA could strengthen bulb dormancy and inhibit bulb germination in lily. Based on weighted gene coexpression network analysis (WGCNA), we identified *LdXERICO*, a gene significantly associated with ABA. Silencing of *LdXERICO* accelerated the germination of small bulbs, which contrasted with the phenotype of *LdXERICO*-overexpressing lines. In genetic transformation experiments with *Arabidopsis* mutants, we found that the inhibitory effect of *LdXERICO* on seed germination was dependent on the ABA pathway. In addition, *LdXERICO*-silenced lines were more sensitive to low temperature, and the expression of *LdICE1*, a low-temperature response factor highly coexpressed with *LdXERICO*, was also significantly upregulated. Overexpression of *LdICE1* increased seed germination and resulted in decreased expression of *AtXerico* in *Arabidopsis thaliana*. Therefore, we propose that *LdXERICO* maintains seed dormancy through the ABA metabolic pathway and that its expression may be decreased by the temperature response factor LdICE1, leading to the release of dormancy.

## Results

### Exogenous abscisic acid affects lily bulb germination

**Figure 1:**
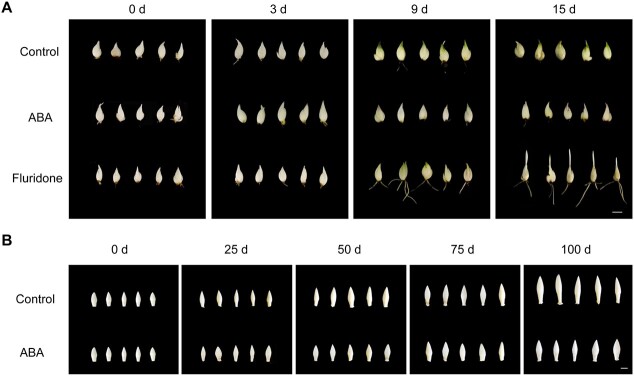
Lily bulb bud and bulblet dormancy release were affected by ABA and fluridone. (A) Phenotypes of bulblets treated with exogenous ABA (25 μM) and fluridone (25 μM) for 2 hours and grown at room temperature (25°C) for 15 days. (B) Phenotypes of lily bulb buds with a circumference of 12–14 cm treated with exogenous ABA (200 mg/l) for 12 hours and stored at low temperature (4°C) for 100 days. Scale bars = 1 cm.

In our preliminary experiments, we found that the ABA content in the buds of dormant *L. davidii* var. *unicolor* was extremely high and decreased sharply with dormancy release [4]. Herein we quantified the endogenous ABA content in the scales, and the results showed that the content decreased with the relief of bulb dormancy, and that the content was very low (Supplementary Data Fig. S1), which further confirmed that the release of dormancy in *L. davidii* var. *unicolor* was mainly dependent on the degradation of ABA in the buds. To determine the effect of ABA on lily dormancy, we treated bulbs of *L. davidii* var. *unicolor* that had a circumference of 12–14 cm and bulblets that had a circumference of 2–3 cm by embedding the scales with exogenous ABA and an ABA inhibitor (fluridone). The results showed that the buds of the bulbs not treated with ABA were significantly elongated, and that the sprouting rate reached 100% after 100 days of storage at 4°C, while the buds of the bulbs treated with ABA grew slowly after 100 days of storage and could not germinate normally after they were transplanted into pots. Moreover, after ABA treatment, the buds could not sprout normally after low-temperature storage. As for the bulblets, only a small percentage sprouted at room temperature (25°C) at 15 days after transplanting, and the sprouting rate reached 50% within 1 month. In contrast, the sprouting rate of bulblets increased, and root growth significantly accelerated after treatment with fluridone, while the bulblets treated with ABA did not sprout at all (Fig. 1). Taken together, these results indicated that ABA treatment could promote bulb dormancy and inhibit bulb germination, regardless of the size of the lily bulbs.

### Screening of hub genes involved in the release of dormancy in lily

To explore the key genes involved in the regulation of dormancy in lily and elucidate the mechanism of dormancy, we screened 18 samples (lily buds and scales) by WGCNA (including three biological replicates) based on sampling methods used in our past studies [4]. After removing the non-expressed and weakly expressed genes, we identified a total of 22 359 differentially expressed genes, and 14 gene coexpression modules were constructed via the WGCNA algorithm (Supplementary Data Fig. S2A). The size of these gene modules ranged from 8714 eigengenes (turquoise) to 164 eigengenes (tan). Among them, six modules were significantly related to dormancy release.

**Figure 2:**
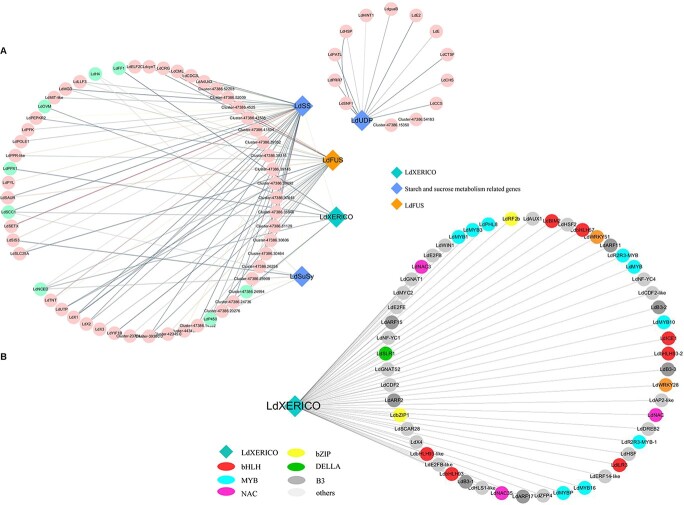
Screening of gene modules based on WGCNA results. (A) Coexpression network of hub genes during dormancy release. The blue–green nodes represent candidate genes and the yellow nodes represent hub genes. The red line indicates a positive correlation and the green line indicates a negative correlation (*P* ≤ .05). (B) TFs highly coexpressed with *LdXERICO*. Nodes of different colors represent members of the same family of TFs (*P* ≤ .05).

To further explore the association between ABA and the network modules, Pearson’s correlation coefficients between module characteristic genes and trait variables were used to represent the correlation strength between modules and metabolites, and a heat map of these findings was constructed (Supplementary Data Fig. S2B). The results showed that the black modules highly correlated with ABA were also highly correlated with the transcripts in buds during dormancy (Supplementary Data Fig. S2C). To further explore hub genes, the differentially expressed genes in the black module were analyzed. According to the weight value, a total of five genes were screened, among which a RING E3 family member, *XERICO*, was identified (Fig. 2A). At the same time, the correlation analysis between XERICO and previous candidate differentially expressed transcription factor (TF)-encoding genes (*P* ≤ .05) showed that a large number of TFs, including bHLH, MYB, WRKY, NAC, and bZIP family members, were closely related to *XERICO*. Among these TFs, ICE1 of the bHLH family was found to be highly correlated with *XERICO* (Fig. 2B).

### Identification of *LdXERICO*s

Numerous *XERICO* genes have been isolated and identified in different plant species, including monocotyledonous and dicotyledonous species, and most of these genes contain a RING-H2 domain [21–23]. Based on the WGCNA results, *LdXERICO*, a key gene that is significantly differentially expressed in the process of dormancy release and that is highly correlated with endogenous ABA, was identified in this study. *LdXERICO* encodes a protein similar to a RING-type E3 ligase protein comprising 134 amino acids. XERICO proteins, such as LdXERICO and AtXERICO in *Arabidopsis*, ZmXERICO in maize, DcXERICO in *Dendrobium officinale* and ZoXERICO in ginger, and their typical RING-H2 structures are shown in Supplementary Data Fig. S3A. The structures of these proteins indicate that the RING-H2 domain is highly conserved across different species (all protein sequences are shown in Supplementary Data Table S1). However, the sequence of the LdXERICO protein was greatly different from that of AtXERICO (<35% similarity). By constructing a LdXERICO protein phylogenetic tree, we found that lily LdXERICO proteins clustered together in the same branch as XERICO proteins in most monocotyledonous plant species, suggesting that they are closely related and/or have similar functions (Supplementary Data Fig. S3B). Therefore, it is of great biological significance to explore the function of the *LdXERICO* gene to elucidate the molecular mechanism of dormancy in various plant species.

### The LdXERICO-GFP fusion protein targets the nucleus in tobacco leaves

**Figure 3:**
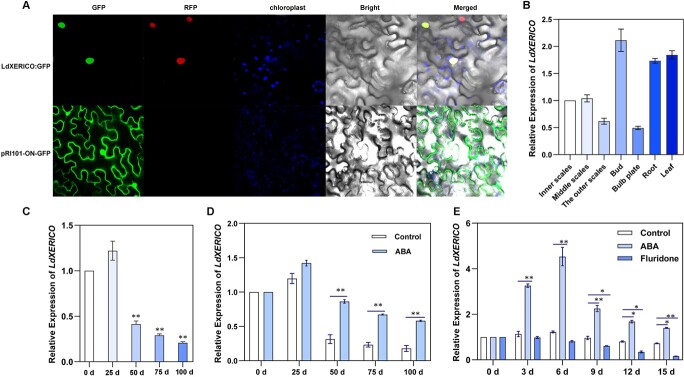
Subcellular localization and expression pattern of *LdXERICO*. (A) LdXERICO subcellular localization. Nuclear marker, positive control; GFP, green fluorescence channel; Merged, mixed field, which indicates superimposed images; Bright, bright field. (B) Expression of *LdXERICO* in different lily tissues. (C) *LdXERICO* expression at 0, 25, 50, 75, and 100 days of dormancy. (D) Expression of *LdXERICO* in buds after ABA treatment (200 mg/l) at 0, 25, 50, 75, and 100 days of dormancy. (E) *LdXERICO* expression in bulblets after exogenous ABA (25 μM) and fluridone (25 μM) were added for 0, 3, 6, 9, 12, and 15 days. The data shown represent three independent biological replicates and three technical replicates (*n* = 3). ^*^P < .05, ^**^P < .01.

The sequence of the LdXERICO protein was analyzed using transmembrane domain prediction software (TMHMM2.0), and it was found that LdXERICO is unlikely to contain a transmembrane region. In contrast, both ZmXerico1 and ZmXerico2 have a transmembrane domain. Further studies showed that both are localized in the endoplasmic reticulum of maize [20]. Therefore, LdXERICO-GFP was introduced into tobacco leaves to determine the subcellular localization of the LdXERICO protein according to previous methods [60]. We transiently expressed this fusion construct in tobacco leaf epidermal cells, which we then evaluated via confocal microscopy imaging. The results showed that the fusion proteins of the LdXERICO and pRI101-ON-GFP vectors were expressed only in the nucleus, consistent with the localization of plant markers, indicating that LdXERICO was localized in the nucleus (Fig. 3A).

### *LdXERICO* was differentially expressed during dormancy release in lily and responded to abscisic acid

Here we used bud, inner scale, middle scale, outer scale, bulb disc, root, and leaf tissues as samples to analyze the tissue-specific expression of *LdXERICO* via qRT–PCR. We found the highest expression levels in the buds and leaves, followed by the basal roots, suggesting that *LdXERICO* is widely expressed in *L. davidii* var. *unicolor*, and that its transcript accumulated more in the major growth points of the lily bulb (Fig. 3B). Then, we examined the native expression pattern of *LdXERICO* in buds during the lily dormancy release process. Using qRT–PCR, we tracked *LdXERICO* expression after 0, 25, 50, 75, and 100 days. The data showed that *LdXERICO* expression was slightly upregulated after 25 days at 4°C. With prolonged exposure to low temperature, *LdXERICO* expression significantly decreased (Fig. 3C). These results indicated that *LdXERICO* can respond to temperature signals and participates in the process of dormancy release in lily.

In previous studies, *PtXERICO* expression in poplar was thought to be closely related to ABA and was significantly upregulated under exogenous ABA treatment [23]. We found that *LdXERICO* could rapidly respond to exogenous ABA and was significantly upregulated after 90 minutes of ABA treatment (Supplementary Data Fig. S4). The results showed that *LdXERICO* expression in bulbs treated with exogenous ABA tended to be downregulated under low-temperature storage. However, compared with the expression of the controls in the same period, the expression of *LdXERICO* in ABA-treated bulbs significantly increased by 1–2 times, and the upregulation of *LdXERICO* was most significant after 50 days of ABA treatment (Fig. 3D). For bulblets, *LdXERICO* expression tended to be downregulated at room temperature within 15 days after transplanting. After 3 days of ABA treatment, *LdXERICO* was rapidly upregulated by >3 times and peaked after 6 days. Although *LdXERICO* expression was downregulated in the later period, its expression was still significantly higher than that in the controls after 15 days. However, the *LdXERICO* transcription level decreased in bulblets treated with fluridone, especially at 15 days after transplanting (Fig. 3E).

### Silencing *LdXERICO* increased sensitivity to low temperature and accelerated sprouting of lily bulbs

**Figure 4:**
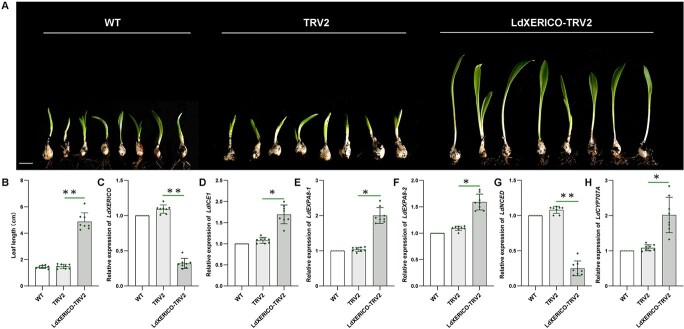
Silencing *LdXERICO* promotes lily bulblet sprouting. (A) Sprouting phenotypes of controls and gene-silenced lines at 14 days. Scale bar = 1 cm. (B) Leaf length statistics of silenced lines. (C) *LdXERICO* expression in silenced lines. (D–H) *LdICE1*, *LdEXPA8-1*, *LdEXPA8-2*, *LdNCED*, and *LdCYP707A* expression in silenced lines. The data are shown as three independent biological replicates and three technical replicates (*n* = 3). ^*^P < .05, ^**^P < .01.

In the past decade, an increasing number of *XERICO* genes have been identified in different species, and most of their functions have been reported to be involved in plant stress response processes, including under drought and salt stress [21, 22, 24]. However, there has been no direct biological evidence of *XERICO* playing a role in dormancy, especially in bulbous plants. To address the role of *LdXERICO* in dormancy release, we silenced *LdXERICO* in bulblets after a period of low-temperature storage. The silenced lines were more sensitive to low temperature and produced leaves nearly four times longer than those of the controls (Fig. 4A and B). *LdXERICO* expression was significantly decreased in the eight silenced lines (Fig. 4C). Conversely, the expression of the low temperature-induced factor *LdICE1* was increased in the silenced lines (Fig. 4D). We also measured the expression of *EXPA8*, a key gene involved in cell expansion. *EXPA*s are among the key downstream genes involved in seed germination, and they promote seed germination by mediating the gibberellic acid (GA) signal transduction pathway [25]. Previous work revealed two *EXPA8*s, both of which were significantly upregulated in the process of dormancy release [4]. The results of this experiment showed that the transcription levels of *LdEXPA8-1* and *LdEXPA8-2* were significantly lower than those in the controls (Fig. 4E and F). The ABA biosynthesis rate-limiting enzyme gene *LdNCED* was downregulated compared with that of the controls (Fig. 4G), while the expression of the *LdCYP707A* gene encoding 8′-hydroxylase in the ABA metabolism pathway was upregulated (Fig. 4H). These results suggested that silencing *LdXERICO* increased the sensitivity of bulblets to low temperature, inhibited ABA accumulation, and accelerated lily sprouting.

### Overexpression of *LdXERICO* delayed germination of *Arabidopsis thaliana* and functioned synergistically with abscisic acid

**Figure 5:**
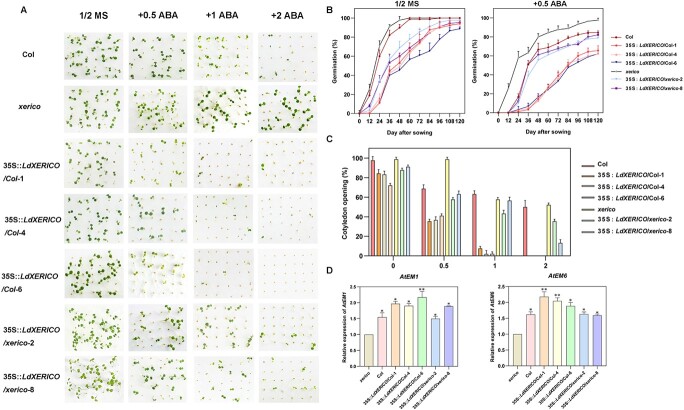
*LdXERICO* inhibited seed germination and induced downstream gene expression in response to ABA. (A) Transgenic *Arabidopsis* seeds overexpressing *LdXERICO* are sensitive to exogenous ABA. Seeds of Col, *xerico*, *LdXERICO* overexpression lines (35S::*LdXERICO*/Col-1, 35S::*LdXERICO*/Col-4 and 35S::*LdXERICO*/Col-6) and genetic complement lines (35S::*LdXERICO*/*xerico*-2 and 35S::*LdXERICO*/*xerico*-8) were sterilized and vernalized. Subsequently, the samples were sown evenly onto filter paper soaked with 1/2-strength MS, 1/2-strength MS +0.5 μM/l ABA, 1/2-strength MS + 1 μM/l ABA, and 1/2-strength MS + 2 μM/l ABA, and images were taken 5 days later. (B) Germination rate statistics of Col, *xerico* and transgenic seeds. The seeds were observed every 12 hours for 120 hours, and the germination rate was calculated. (C) Cotyledon opening of *A. thaliana*. The treatment methods of Col, *xerico* and transgenic seeds were the same as above, and statistical data were recorded and calculated every 24 hours. (D) Expression of *AtEM1* and *AtEM6* in Col, *xerico*, and transgenic lines. The data are shown as three independent biological replicates and three technical replicates. ^*^P < .05, ^**^P < .01.

To further investigate the function of *LdXERICO*, we ectopically expressed 35S::*LdXERICO* in *xerico* mutants and wild-type (Col) *Arabidopsis*. In the absence of ABA, there was no significant difference in germination rates between the wild-type (Col) and 35S::*LdXERICO*/Col lines, but for the *xerico* mutant the germination rate reached 100% at 60 days (Fig. 5A). When 0.5 μM ABA was added, the germination rates of the wild-type and mutant seeds were slightly reduced, but >60% of seeds still germinated, while the seeds of the 35S::*LdXERICO*/Col lines showed increased ABA sensitivity. After 24 hours of treatment, the germination rate was still 0% (Fig. 5B). We then increased the ABA concentration to 1 μM, and the seedlings of 35S::*LdXERICO*/Col lines showed more severe growth retardation, while two complemented lines showed reduced sensitivity to exogenous ABA. More salient results were observed with the 2 μM ABA treatment (Fig. 5B). At 5 days after sowing, we calculated the cotyledon opening rate for *A. thaliana*. In 1/2-strength Murashige and Skoog (MS) medium supplemented with 1 μM ABA, the 35S::*LdXERICO*/Col lines hardly developed cotyledons, while ~30% of the complemented lines could still develop complete cotyledons (Fig. 5C). Using qRT–PCR analysis, we found that the transcript levels of *AtEM1* and *AtEM6* were upregulated to various degrees in 35S::*LdXERICO*/Col lines but decreased in the complemented lines (Fig. 5D). These results indicated that *LdXERICO* could inhibit seed germination and had a synergistic effect with ABA.

### Overexpression of *LdXERICO* delayed growth and repressed bulblet development by strengthening the degree of dormancy in lily

**Figure 6:**
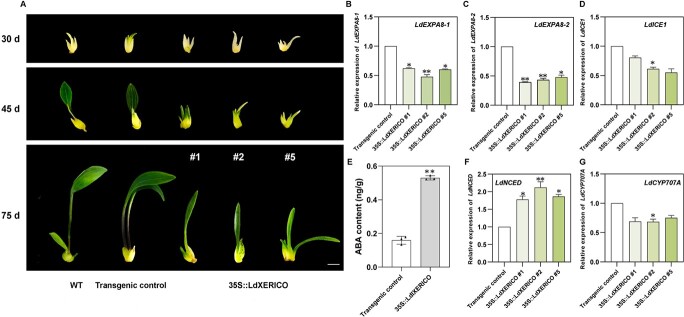
*LdXERICO* inhibits the sprouting and growth of lily bulblets. (A) Phenotypes of controls and transgenic bulblets at 30, 45, and 75 days under tissue culture. Scale bar = 1 cm. (B, C) Expression of *LdEXPA8-1* and *LdEXPA8-2* in transgenic lines. (D) Expression of *LdICE1* in transgenic lines. (E) ABA content in transgenic lines. (F, G) Expression of *LdNCED* and *LdCYP707A* in transgenic lines. The data are shown as three independent biological replicates and three technical replicates. ^*^P < .05, ^**^P < .01. Scale bar = 1 cm.

As described above, we speculated that lily bulb dormancy may be regulated by *LdXERICO*. To test our hypothesis, we overexpressed the *LdXERICO* gene in ‘White Paradise’ lily via a stable and efficient genetic transformation system developed in an earlier study by our team [26]. The scales of transgenic lines and controls were cut to induce bulblet development under dark conditions. When the bulblets grew to the same size, they were subjected to a normal photoperiod (16/8 hours light/darkness) and cultured for morphological comparisons. After 15 days of growth, the controls began to sprout and produce leaves, while the transgenic lines began to turn green after 30 days. The observations were continued for 50 days. Compared with those of the controls, the bulblets of transgenic lines exhibited slower sprouting and smaller leaf area (Fig. 6A). LdEXPA8-1 and LdEXPA8-2 showed lower levels in the transgenic lines than in the controls (Fig. 6B and C). In contrast with the results when silencing the *LdXERICO* gene, the expression of *LdICE1* in the transgenic lines was lower than that in the controls (Fig. 6D). The ABA content in the transgenic lines was significantly higher than that in the control (Fig. 6E). Correspondingly, *LdNCED* was significantly upregulated in the transgenic lines compared with the controls (Fig. 6F), and *LdCYP707A* expression showed the opposite trend (Fig. 6G). These findings suggested that *LdXERICO* may promote ABA accumulation, which then may inhibit the sprouting of lily bulbs.

### *LdXERICO* expression may be downregulated by the low temperature-induced factor LdICE1

**Figure 7:**
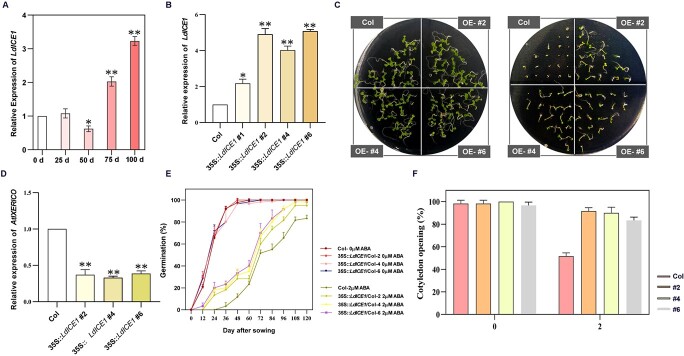
LdICE1 promotes seed germination and its overexpression results in decreased expression of *AtXERICO*. (A) *LdICE1* expression at 0, 25, 50, 75, and 100 days of dormancy release. (B) Expression of *LdICE1* in four *LdICE1*-overexpressing lines. Expression of *LdICE1* in overexpressing *A. thaliana* and phenotype of transgenic lines. (C, E, F) Seed germination rate and cotyledon opening of transgenic *A. thaliana* in medium supplemented with 2 μM/l ABA were statistically analyzed, and the data are representative of three technical replicates. (D) Expression of *AtXERICO* in *LdICE1*-overexpressing lines. The data are representative of three independent biological replicates and three technical replicates. ^*^P < .05, ^**^P < .01.

ICE1 is a key TF that functions in response to low-temperature signaling in *Arabidopsis*, and mutants lacking *ICE1* exhibit delayed germination [27]. In this study, *LdICE1* and *LdXERICO* were highly coexpressed. With the release of dormancy by storage at 4°C, *LdICE1* showed an overall trend of significant upregulation, and its expression increased to nearly 3-fold higher at 100 days (Fig. 7A). The germination rate of transgenic *A. thaliana* seeds was significantly higher than that of Col seeds when 2 μM ABA was added. Continuous observations of cotyledon opening revealed that more than half of Col plants failed to develop cotyledons. However, >80% of transgenic lines could still develop complete cotyledons (Fig. 7E and F). At the same time, *AtXERICO* expression in the transgenic lines was significantly downregulated compared with that in Col (Fig. 7D), which contrasted with the expression trend of *LdICE1* in *LdXERICO*-silenced lines (Fig. 4D). Based on the above results, we hypothesized that LdICE1 plays a regulatory role as an upstream regulator of *LdXERICO*.

## Discussion

### Effects of abscisic acid on bulb dormancy

Various hormone signal transduction pathways are important factors in regulating bulb dormancy and sprouting [28–30]. ABA is one of the most important hormones in plants and is involved in many physiological processes, including dormancy, germination, fruit ripening, and plant responses to various stresses [28, 31–33]. In previous research we concluded that ABA degradation was one of the key factors determining the release of dormancy in lily by quantitative detection of various endogenous hormones [4]. In the last decade exogenous ABA treatment has provided more evidence in investigations of whether and how ABA affects dormancy [16, 34]. In some woody plant species the application of exogenous ABA strengthens plant dormancy and delays bud germination [12, 31, 35]. In addition, several studies have shown that the location and timing of exogenous application of ABA seemed to be more critical, and the application of ABA on the leaf surface before germination had a slight promoting effect on the flowering of dormant peach trees [36]. In our study, exogenous ABA treatment significantly strengthened the degree of dormancy in lily bulbs and bulblets (Fig. 1). However, this phenomenon was alleviated by treatment with fluridone, which accelerated the sprouting of bulblets and significantly promoted root growth (Fig. 1B). It has been shown that fluridone can increase the germination rates of red rice [34], rapeseed [37], and ryegrass [38] without reducing endogenous ABA levels, and continued application of ABA could not counteract the effect of fluridone [39, 40], suggesting that ABA is the key hormone controlling bulb dormancy in lily.

### *LdXERICO* is a key gene involved in the regulation of bulb dormancy

Lily dormancy is a complex physiological process involving a wide range of metabolic activities and a vast gene regulatory network [3, 4, 7]. The functions of traditional ABA metabolism- and signal transduction-related genes such as *NCED* [41, 42], *CYP707A *[41, 43, 44], ABI5 [27, 45], and PP2C [46, 47] in plant dormancy have been widely reported. In this study, the key regulatory role of ABA in dormancy was clarified, and a gene coexpression network was constructed based on WGCNA. A RING-type ubiquitin ligase gene, *LdXERICO*, was ultimately identified (Supplementary Data Fig. S2). At present, many *XERICO* genes have been cloned and identified in >100 plant species, including dicotyledonous and monocotyledonous species. However, there are some differences in the expression patterns of *XERICO* in different tissues of different species. *ZmXERICO* in maize was expressed in most tissues but was expressed at significantly higher levels in roots, leaves, and pistils than in other tissues [48], whereas poplar *PtXERICO* was abundantly expressed in stems and early shoots [23]. We found that *LdXERICO* transcription levels increased significantly more at the main point of growth (Fig. 3B). The expression of *LdXERICO* in buds was briefly induced in the early stage of 4°C storage and then decreased sharply, reaching the lowest value at 100 days (Fig. 3C). Previous studies have shown that drought stress, NaCl, ABA, and cold treatment can strongly induce *XERICO* expression [20–22, 48]. However, our experiments showed that decrease in temperature accelerated the downregulation of *LdXERICO* gene expression. The changes in endogenous ABA content in buds were basically consistent with the expression trend of *LdXERICO*, indicating that *LdXERICO* had a synergistic effect with ABA. The results of exogenous treatment experiments further supported our view, and *LdXERICO* expression was always significantly higher after ABA treatment than in the controls (Fig. 3D). The above findings may indicate that the stress response to abiotic stress occurred in the bulb. However, with the decrease in temperature, the transcription level of *LdXERICO* may be inhibited by temperature response factors, and the induction effect of exogenous ABA is also cancelled.

### *LdXERICO* negatively regulates bulb dormancy release

In eukaryotic cells, E3 ligase, as an important member of the ubiquitin proteolytic system, plays an important role in determining the specificity of substrate proteins, regulating cells and maintaining their basic functions [49, 50]. Several E3 members were confirmed to be involved in seed germination, flowering, and response to temperature [51–53]. Here we found that *LdXERICO*, a member of the RING-H2 type E3 ligase family, is involved in lily dormancy release. Most previous reports on *XERICO* function have focused on responses to abiotic stress. Overexpression of *AtXERICO*, *ZmXERICOs*, and *PtXERICO* in *Arabidopsis*, maize, and poplar resulted in increased drought tolerance [20, 21, 23, 24]. When *AtXERICO* was overexpressed in rice, the leaf transpiration and water consumption of transgenic rice were lower than those of the wild type, and the drought tolerance of transgenic rice was significantly improved [22]. This study is the first to investigate the role of *LdXERICO* in the process of dormancy release in lily by low temperature. In *LdXERICO*-silenced lines, bulbs sprouted rapidly after potting, and the leaf length was more than twice that of the controls after 15 days (Fig. 4A). Overexpression of the *LdXERICO* gene in *xerico* mutants delayed seed germination, and transgenic *Arabidopsis* was sensitive to ABA treatment (Fig. 5A). Subsequently, we also carried out stable genetic transformation of *LdXERICO* in lily, and the results showed that the transgenic lines had delayed sprouting, slow leaf development, and smaller leaf area (Fig. 6A). Therefore, we conclude that *LdXERICO* inhibits the sprouting of lily and plays a similar role in leaf growth and morphogenesis during dormancy release. Previous studies have shown that the *XERICO* gene is involved in plant resistance to various abiotic stresses [21–24]. In this study we measured the endogenous ABA content in overexpressing lines, and the results showed that ABA content in overexpressing lines was higher than that in controls (Fig. 6D). We also found that *LdNCED* and *LdCYP707A* were significantly differentially expressed in both silenced and transgenic lines. Under drought stress, the expression levels of *AtNCED3*, *OsNCED*, *OsABA3*, *OsABI5*, and *OSLEA3-1-1* in *AtXERICO* transgenic *A. thaliana* and rice plants were significantly higher than those in wild-type plants. The findings of this study also suggested that *AtXERICO* may improve the drought tolerance of plants by regulating the ABA biosynthesis pathway [21, 22]. In maize, *ZmXerico* affected ABA signaling by regulating the stability of 8′-hydroxylase, ultimately increasing the ABA content, which further confirmed the above interpretation [20]. In contrast to the results in maize, we found no transmembrane domain in the LdXERICO protein, and the subcellular localization results showed that LdXERICO was indeed localized in the nucleus. We speculate that the LdXERICO protein does not directly regulate the stability of ABA 8′-hydroxylase. This view was corroborated by the results of our experiments. In this study, we identified a gene encoding a protein with similarity to ABA 8′-hydroxylase named LdCYP707A, which was significantly upregulated during dormancy release (Supplementary Data Fig. S5A and C). The subcellular localization showed that LdCYP707A localized in the endoplasmic reticulum (Supplementary Data Fig. S5D). However, bimolecular fluorescence complementation (BiFC) tests did not reveal protein–protein interactions between LdXERICO and LdCYP707A. Thus, *LdXERICO* may promote endogenous ABA content through other mechanisms in lily.

**Figure 8:**
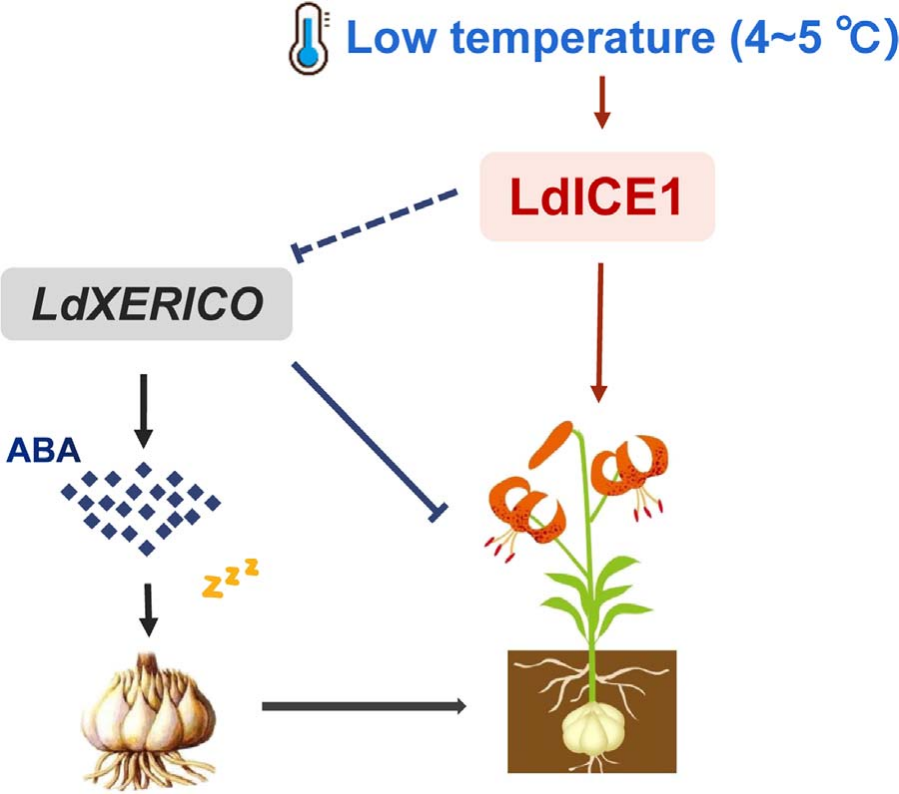
Model of lily bulb dormancy release regulated by *LdXERICO*. *LdXERICO* promoted the maintenance of dormancy in lily bulbs by increasing endogenous ABA content. In the process of releasing lily bulb dormancy caused by low temperature, LdICE1 has a negative effect on *LdXERICO* expression, leading to the downregulation of *LdXERICO* expression and ultimately promoting bulb sprouting.

Although there is increasing evidence that *XERICO* plays a key role in the regulation of plant growth and development through the ABA pathway, the upstream regulators of *XERICO* are poorly explored. In *Arabidopsis*, *XERICO*, as a downstream target gene of DELLA, is positively regulated by DELLA and promotes the accumulation of endogenous ABA, which plays an important role in a variety of physiological activities [54]. Overexpression of the GA receptor *GID1b* rescues the delayed germination phenotype of *sly1-2* mutants during seed maturation in *Arabidopsis* and leads to downregulation of DELLA and decreased ABA content, apparently as a result of reduced *XERICO* transcription levels [55]. In recent studies, AtCBF4 was shown to play an inhibitory role upstream of *AtXERICO*, which attenuates ABA signaling and the plant response to stress [24]. On the basis of the WGCNA results, we screened a large number of TF family members, including the C-REPEAT BINDING FACTOR (CBF)-induced gene *LdICE1*, which was highly coexpressed with the *LdXERICO* gene and significantly upregulated in the process of dormancy release. ICE1 directly activates the expression of cold-responsive CBF, which plays a crucial role in the cold signal response in plants [56]. It is well known that lily bulbs can grow and flower normally only after a certain period of low-temperature storage [4]. Here we found that the expression of *LdICE1* in *LdXERICO*-silenced lines was significantly higher than that in controls, and bulblets were more sensitive to low temperature and germinated earlier (Fig. 4D). Meanwhile, we also found that the overexpression of *LdICE1* in *Arabidopsis* increased seed germination and decreased plant sensitivity to ABA (Fig. 7C). The expression of *AtXERICO* in *LdICE1*-overexpressing lines was significantly decreased (Fig. 7D). Interestingly, the expression of *LdICE1* showed the opposite trend in *LdXERICO*-overexpressing lines (Fig. 6D). The *ice1* mutant exhibited delayed germination and plant albinism. Moreover, AtICE1 inhibits *ABI5* and *DELLA* activity and forms a circular regulatory network to maintain appropriate levels of ABA signaling during *Arabidopsis* seed germination [27]. Therefore, we speculate that *LdICE1* may be upstream of *LdXERICO* and that there may be a regulatory relationship between LdXERICO and the LdICE1 protein. The transcript level of *LdICE1* increased under a continuous low-temperature signal, which accelerated dormancy release by inhibiting the expression of *LdXERICO* (Fig. 8).

## Materials and methods

### Plant materials

According to the results of previous studies, bulbs of *L. davidii* var. *unicolor* were collected in October 2019, 2020, and 2021 [4]. The materials were single-headed bulbs that were of the same size (12–14 cm in circumference), had a similar weight (30 ± 2 g), and were free of pests and diseases. For tissue-specific analysis, we selected inner scales, middle scales, the outer scales, bud, bulb plate, roots, and leaf of the same plant as materials to determine the expression of the *LdXERICO* gene. Coconut bran with a good structure and a 70–75% water content was used as the storage substrate, and the bulbs were stored at 4°C. After 0, 25, 50, 75, and 100 days of low-temperature (4°C) storage, the buds were removed, flash-frozen in liquid nitrogen, and stored at −80°C. In addition, lily bulbs that were free of disease and insect pests, had tightly held scales, and had a healthy and bright appearance were selected as breeding materials. One hundred fifty scales were selected for each group, and the scales were washed with distilled water. The outer and middle scales were soaked in 1200X carbendazim wettable powder for 30 minutes and then placed at room temperature to remove the surface moisture (relative humidity 50%, temperature 25°C). The prepared matrix (70% moisture) was placed into the bottom 3–5 cm of a plastic basket, and the scales were evenly placed on the matrix, with slight spacing between the scales to allow full contact with the matrix. After 3 months of being embedded in a 25°C (±0.5°C), dark, constant-temperature incubator, bulblets of similar size and mass with a circumference of 2–3 cm were selected for subsequent tests. Three biological replicates were included for each group. The material used for stable genetic transformation of lily in this study was bulblets generated from ‘White Paradise’ tissue culture. The bulblets were stored in a constant-temperature incubator (25 ± 0.5°C, 16/8 hours light/darkness).

The *A. thaliana* plants used for generating transgenic plants in this study were wild-type Columbia-0 (Col-0) plants. *Xerico* (SALK_203161) materials were obtained from the *Arabidopsis* mutant resource platform [The Arabidopsis Information Resource (TAIR); https://www.arabidopsis.org/]. The *Arabidopsis* seeds were sterilized using an aqueous solution containing 30% (v/v) sodium hypochlorite (NaClO) for 4 minutes and subsequently washed five times with sterile water. The seeds were sown in sterile substrate and incubated in a constant-temperature incubator (16/8 hours light/darkness and 68% relative humidity). Tobacco plants were grown under the same conditions.

### Effects of abscisic acid and abscisic acid synthesis inhibitors on dormancy

Dormant bulbs were immersed in 200 mg/l ABA solution and distilled water for 1 hour each. After the surface moisture was removed, the bulbs were stored at 4°C, with coconut bran (70% moisture) used as the storage substrate. The bulblets were soaked in ABA (25 μM), fluridone (inhibitor of ABA biosynthesis, 25 μM), and deionized water for 90 minutes. Then, the bulblets were planted under 16/8 hours (light/dark) conditions at 25°C (± 0.5°C). Samples were collected at 0, 3, 6, 9, 12, and 15 days to calculate the germination rate. Each experiment was repeated three times (*n* = 3).

### Weighted gene coexpression network analysis

In this study, WGCNA was performed to cluster genes with similar expression patterns into the same module, and key differentially expressed genes were screened from among the main modules [57]. On the basis of excluding non-expressed and weakly expressed genes, the correlation coefficients between genes were transformed by a weighting function to form an adjacency matrix, the similarity of overlapping matrices of different topologies was calculated by using an adjacency matrix, and a WGCNA network was constructed by using the R language package (R version 4.0.3). Then, the correlation between modules and phenotypes was analyzed, and Cytoscape software was used to visualize the gene connectivity in the modules to screen out the key differentially expressed genes with high connectivity.

### Protein bioinformatic analysis, phylogenetic analysis, and sequence alignment

The homology of LdXERICO amino acid sequences was compared using NCBI local BLAST searches and DNAMAN 7.0. A protein phylogenetic tree for LdXERICO and LdCYP707A was constructed with MEGA 7 and 1000 bootstraps.

### Gene cloning, vector construction, and gene transformation

The full-length sequence of the *LdXERICO* gene was obtained based on lily RNA sequencing data, and specific primers were designed according to the sequence for PCR amplification. The PCR products were inserted into a PMD18-T vector and sequenced. Sequence alignment was performed using DNAMAN 7.0. The *LdCYP707A*, *LdNCED*, and *LdICE1* genes were isolated and cloned using the same method as above. The NdeI and BamHI restriction sites were selected, and *LdXERICO* was cloned into pRI101-ON and pRI101-AN vector plasmids according to the manufacturer’s instructions for an In-Fusion HD Cloning Kit (TaKaRa, Dalian, China). Lily scales of lily and developing floral tissues of *Arabidopsis* were transformed. The specific operation of lily genetic transformation is based on a stable and efficient genetic transformation method established by our team [26, 58]. *Arabidopsis* (the *xerico* mutant and Col-0) were transformed using the floral-dip method [59]. NdeI and SalI were selected to clone LdICE1 into the pRI101-ON vector plasmid for *Arabidopsis* transformation. *T*_3_-generation homozygous seeds were used for *Arabidopsis*, and *T*_2_-generation homozygous bulblets were used for lily. Seeds and bulblets transformed into empty vectors were used as controls in each experiment. BamHI and EcoRI restriction sites were selected, and the products were cloned into a TRV2 vector for transient gene silencing. Primer 5.0 software was used to design the primers used (Supplementary Data Table S2). The GenBank accession numbers of *LdXERICO*, *LdCYP707A*, and *LdNCED* were OQ283206, OQ283204, and OQ283205.

### Subcellular localization of LdXERICO-GFP and LdCYP707A-GFP

NdeI and BamHI restriction sites were used for cloning and subsequent insertion of *LdXERICO*-encoded sequences into the pRI101-ON-GFP vector. In the same way, HindIII and SalI restriction sites were used for cloning and subsequent insertion of *LdCYP707A*-encoded sequences into the pRI101-ON-GFP vector and the primers used for this purpose are shown in Supplementary Data Table S1. Using the nuclear marker and ER marker as a positive control and pRI101-ON-GFP as a negative control, we transformed the vectors into *Agrobacterium tumefaciens* GV3101 and then injected the *A. tumefaciens* into *Nicotiana benthamiana* leaves [9]. After 2–3 days, the leaves were observed by laser confocal fluorescence microscopy (TCSSP8-SE, Leica, Wetzlar, Germany).

### Virus-induced gene silencing in lily

In this study, virus-induced gene silencing (VIGS) was used to transiently silence the *LdXERICO* gene. The experimental procedure was based on the methods of related studies of lily [26, 60] and *Gladiolus* [45, 61], with some modifications. In brief, TRV1, TRV2, and TRV2-LdXERICO vector plasmids were transformed into the *Agrobacterium* GV3101 strain. Equal volumes of MMA [MS medium + 20 mg/l sucrose+10 mM/l 2-(*N*-morpholino)ethanesulfonic acid (MES) + 200 μm/l acetosyringone (AS)] suspension and bacterial solution were mixed together so that the OD600 ranged from 0.8 to 1.0, and the mixture was incubated in the dark for 5 hours. Ultimately, the bulbs were stored at 4°C for 3 months.

### Seed dormancy and germination analysis

For dormancy and germination tests, seeds of each group were sown on filter paper soaked with 1/2-strength MS medium, 1/2-strength MS + 0.5 μM ABA, 1/2-strength+1 μM/l ABA, and 1/2-strength MS + 2 μM ABA liquid medium (pH = 5.8). The seeds were incubated in Petri dishes (16/8 hours light/darkness, 21 ± 1°C) and observed every 12 hours for 120 hours. The germination rate of each group was calculated. At least three biological replicates were evaluated for each experiment (*n* = 3).

### RNA extraction and real-time–PCR

Total plant RNA was extracted using a small-volume extraction kit (Megen, Guangzhou, China), and reverse transcription was performed using a Fast Quant RT Kit (Tiangen, Beijing, China). Primer Premier 5.0 was used to design the primers used, and 1 μmol cDNA was used as the real-time PCR (qRT–PCR) template. A TaqMan Universal SYBR qPCR Master Mix Real-Time PCR system was used for gene quantitative detection, and the quantitative results were analyzed using the 2ΔΔCT method. *LdFP* was used as the reference gene. All primers used are listed in Supplementary Data Table S1. Three technical replicates and three biological replicates were included per group (*n* = 3).

### HPLC–MS/MS analysis of ABA

To detect the change in endogenous ABA content in *LdXERICO*-overexpressing lines, quantitative analysis of ABA was carried out by the ESI-HPLC–MS/MS method. The specific test operation process is described in our previous study [4].

## Acknowledgements

This work was financed by the National Key R&D Program of China (2018YFD1000407), LiaoNing Revitalization Talents Program (XLYC2002052), Shenyang Innovation Program of Seed Industry (21-110-3-12), and the earmarked fund for CARS (CARS-23).

## Author contributions

X.Y.F. and H.M.S. conceived and designed the experiments. X.M.Z. was involved in VIGS and data analysis. L.L.F. was involved in genetic transformation. M.L., Y.Y., and C.X.W. assisted the authors in data collation and analysis. All of the authors read and approved the final manuscript.

## Data availability

The data supporting the findings of this study are available from the corresponding author upon request.

## Conflict of interest

The authors declare no conflicts of interest in the submission of this manuscript.

## Supplementary data

Supplementary data is available at *Horticulture Research* online.

## Supplementary Material

Web_Material_uhad023
