# Supplementary material for: The RING-H2 gene LdXERICO plays a negative role in dormancy release regulated by low temperature in Lilium davidii var. unicolor
Source: Hortic Res. 2023 Feb 20;10(4):uhad030. doi: 10.1093/hr/uhad030 (PMC10548414; doi:10.1093/hr/uhad030)
Supplement: Web_Material_uhad023 [file web_material_uhad023.zip › supplementary figure.docx]

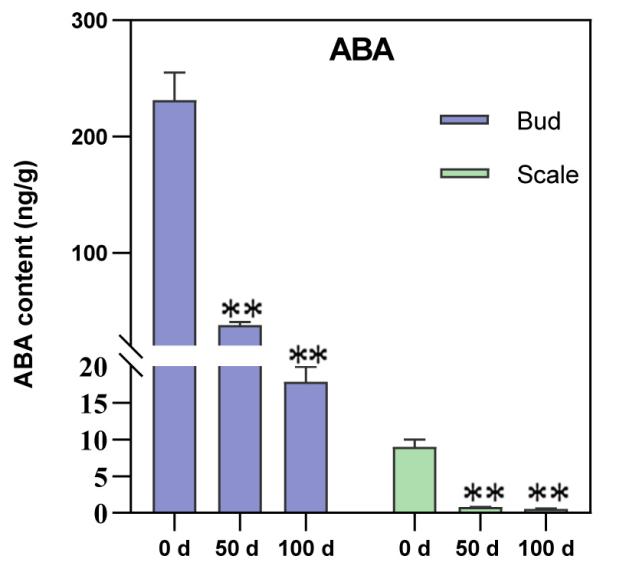


**Supplementary Fig. S1** Changes in endogenous ABA content during low-temperature storage. Changes in endogenous ABA content in buds and scales during bulb dormancy release. Purple represents the ABA level in the bud, and green represents the scale. The data are shown as three independent biological replicates and three technical replicates. **p*<0.05, ***p*<0.01.


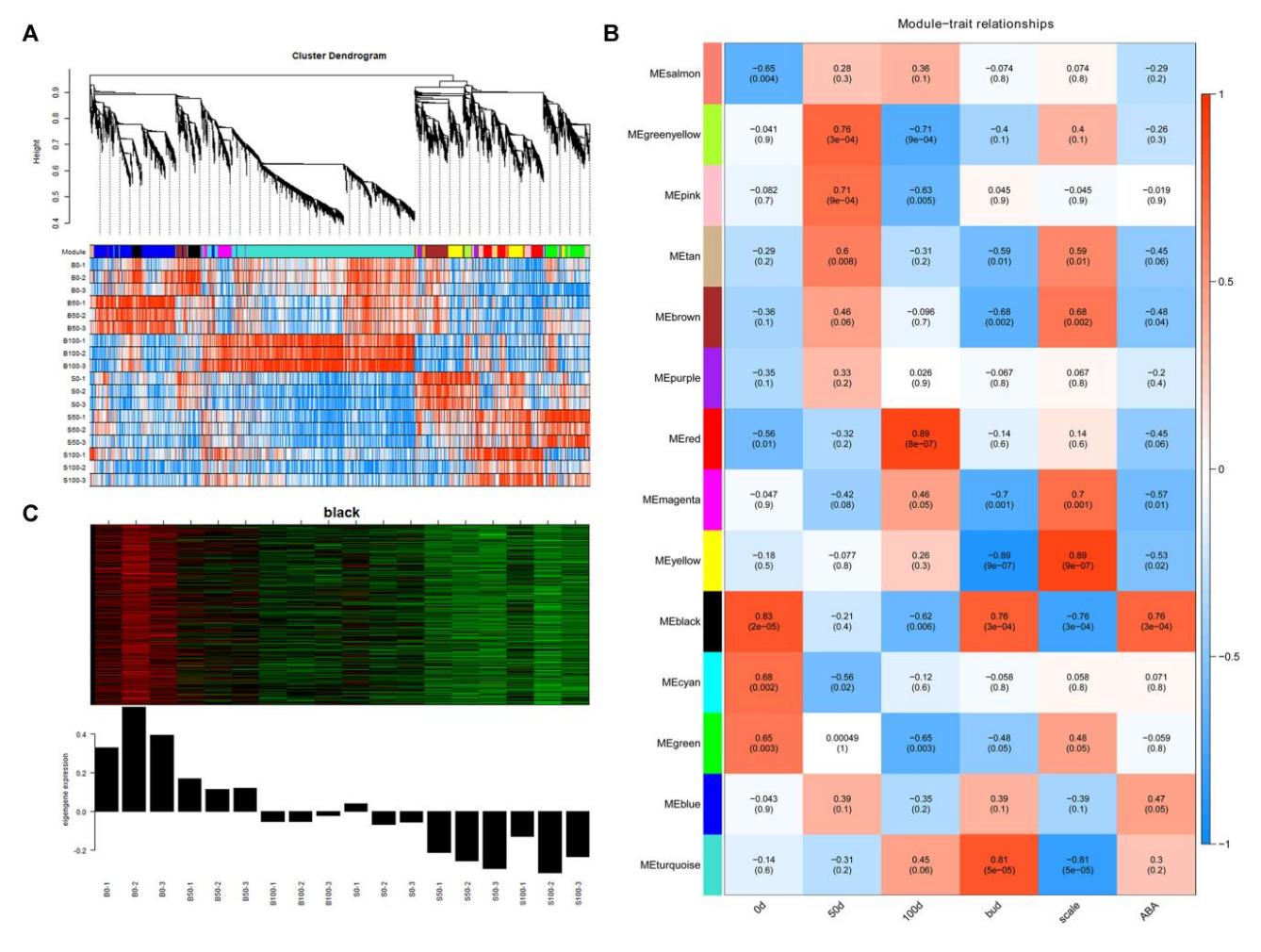


**Supplementary Fig. S2** Screening of gene modules based on WGCNA. **A** WGCNA cluster dendrogram of the 18 sample transcripts. **B** Heatmap of module association with phenotype and endogenous ABA (*P* ≤0.05). **C** Histogram of characteristic gene expression patterns of 18 samples (black module).


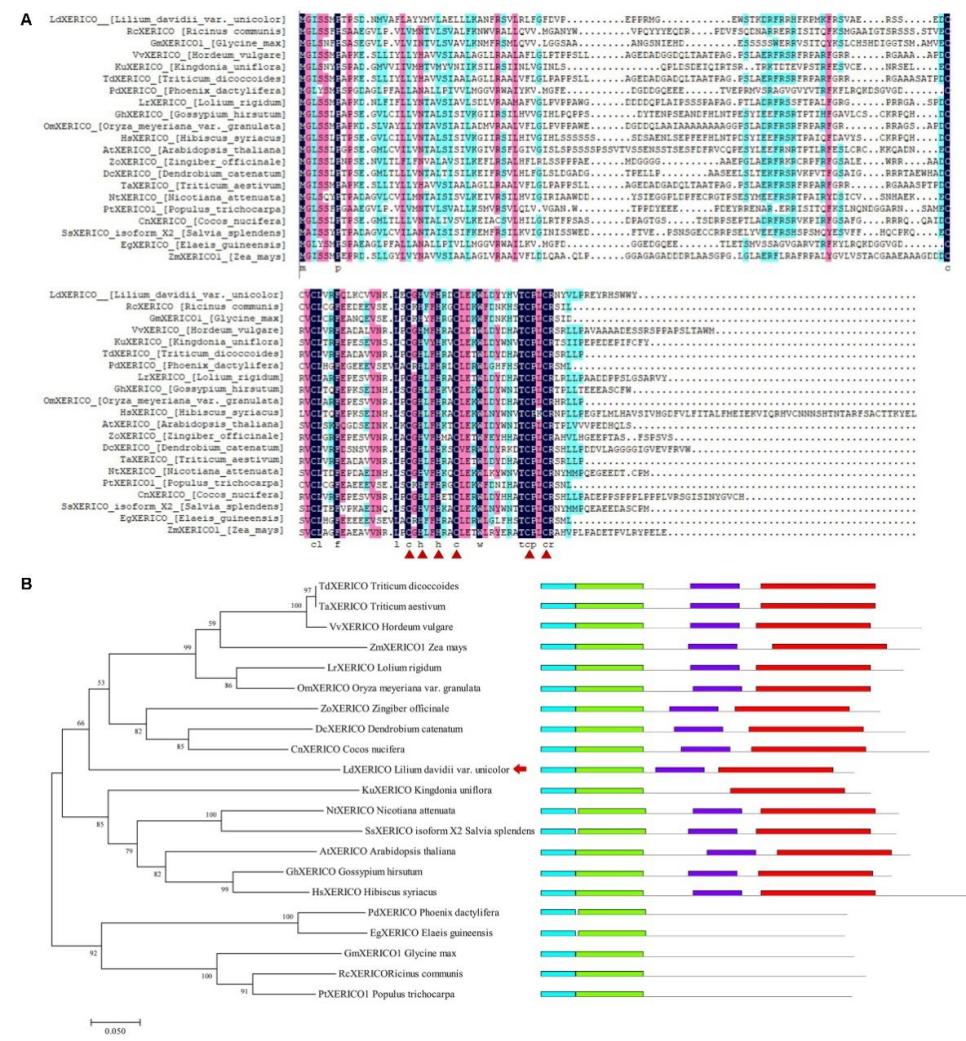


**Supplementary Fig. S3** Identification of *LdXERICO*. **A** Sequence alignment of *Arabidopsis thaliana* (AtXERICO), maize (ZmXERICO1), ginger (ZoXERICO) and other related proteins. The red triangles represents the RING protein family structure domain, the same amino acid in dark blue. **B** Phylogenetic tree construction and conserved motif analysis of the LdXERICO protein.


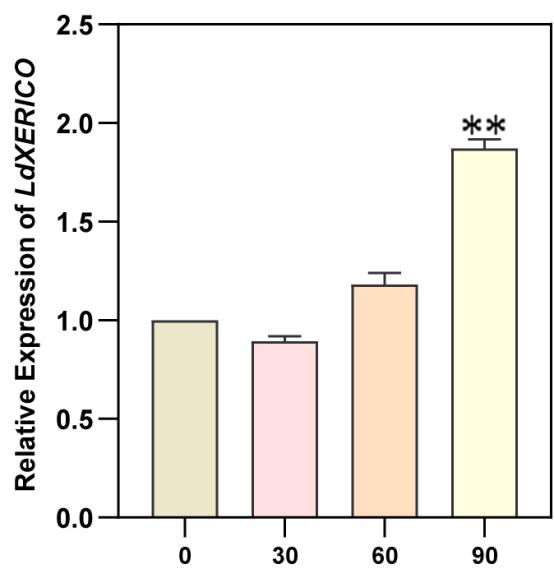


（min）

**Supplementary Fig. S4** Expression pattern of *LdXERICO* under exogenous ABA treatment. *LdXERICO* expression in bulblets after exogenous ABA (25 µM) were added for 0, 30, 60 and 90 min. The data are shown as three independent biological replicates and three technical replicates. **p*<0.05, ***p*<0.01.


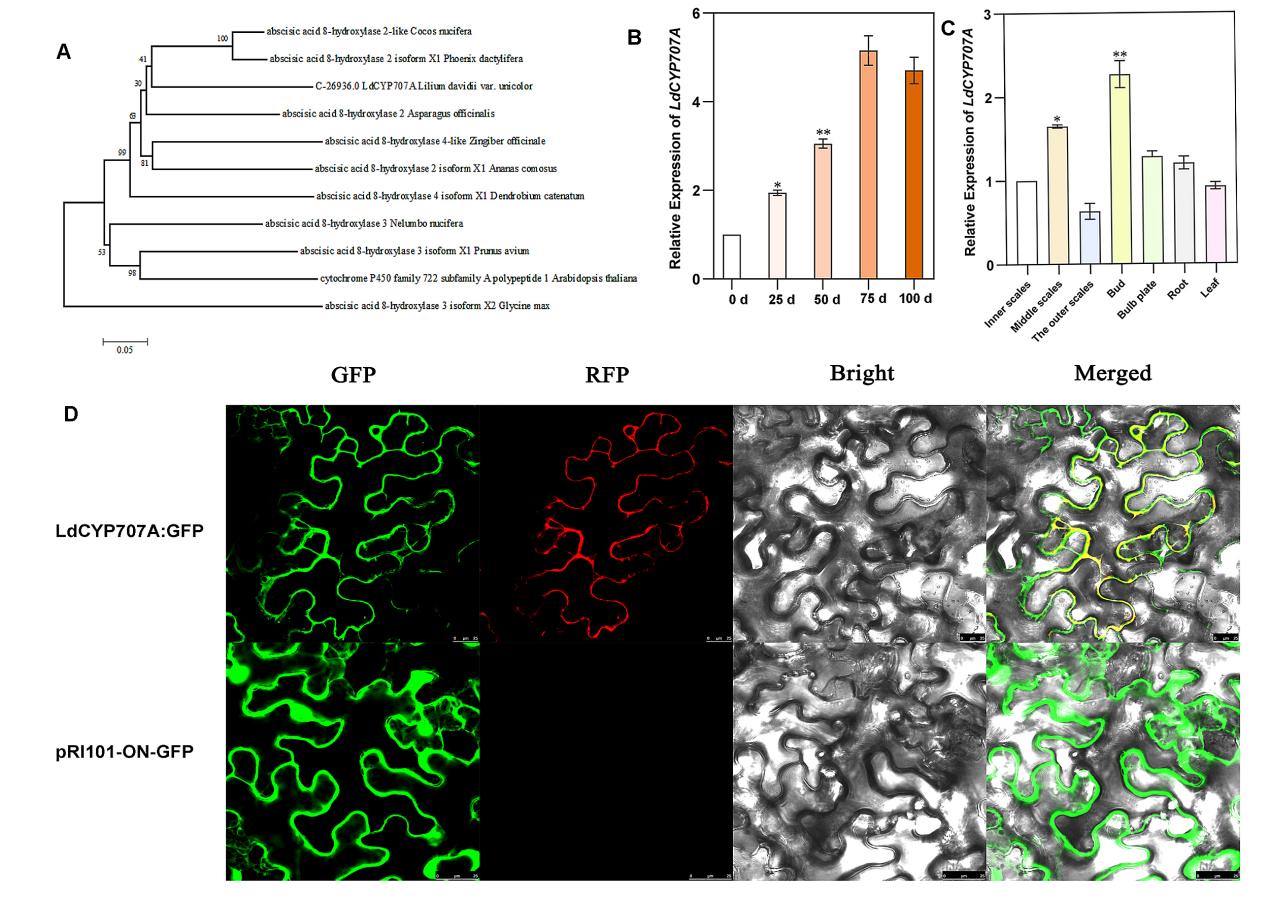


**Supplementary Fig. S5** Identification of *LdCYP707A* and Subcellular localization analysis. A, Phylogenetic tree construction of the LdCYP707A protein. B, *LdCYP707A* expression at 0, 25, 50, 75 and 100 days of dormancy. C, Expression of *LdCYP707A* in different lily tissues. D, LdCYP707A subcellular localization. ER marker: positive control; GFP: green fluorescence channel; Merged: mixed field, which indicates superimposed images; Bright: bright field. **p*<0.05, ***p*<0.01.
